# Heart failure decouples the precuneus in interaction with social cognition and executive functions

**DOI:** 10.1038/s41598-023-28338-0

**Published:** 2023-01-23

**Authors:** Matthias L. Schroeter, Jannis Godulla, Friederike Thiel, Birol Taskin, Frank Beutner, Vladimir K. Dubovoy, Andrej Teren, Julia Camilleri, Simon Eickhoff, Arno Villringer, Karsten Mueller

**Affiliations:** 1 Clinic for Cognitive Neurology, University Hospital Leipzig, Liebigstr. 16, 04103 Leipzig, Germany; 2 Max Planck Institute for Human Cognitive and Brain Sciences, Stephanstrasse 1A, 04103 Leipzig, Germany; 3 Leipzig Research Center for Civilization Diseases, Leipzig, Germany; 4 Ludwig Maximilians University Munich, Munich, Germany; 5 Leipzig Heart Center, Leipzig, Germany; 6 Karazin Kharkiv National University, Kharkiv, Ukraine; 7 Department of Cardiology and Intensive Care Medicine, Klinikum Bielefeld, Bielefeld, Germany; 8 Institute of Systems Neuroscience, Heinrich-Heine University, Düsseldorf, Germany; 9 Institute of Neuroscience and Medicine (INM-7 Brain and Behaviour), Forschungszentrum Jülich, Jülich, Germany

**Keywords:** Cardiology, Heart failure, Neuroscience, Cognitive neuroscience

## Abstract

Aging increases the risk to develop Alzheimer’s disease. Cardiovascular diseases might accelerate this process. Our study aimed at investigating the impact of heart failure on brain connectivity using functional magnetic resonance imaging at resting state. Here we show brain connectivity alterations related to heart failure and cognitive performance. Heart failure decreases brain connectivity in the precuneus. Precuneus dysconnectivity was associated with biomarkers of heart failure—left ventricular ejection fraction and N-terminal prohormone of brain natriuretic peptide—and cognitive performance, predominantly executive function. Meta-analytical data-mining approaches—conducted in the BrainMap and Neurosynth databases—revealed that social and executive cognitive functions are mainly associated with those neural networks. Remarkably, the precuneus, as identified in our study in a mid-life cohort, represents one central functional hub affected by Alzheimer’s disease. A long-term follow-up investigation in our cohort after approximately nine years revealed more severe cognitive impairment in the group with heart failure than controls, where social cognition was the cognitive domain mainly affected, and not memory such as in Alzheimer’s disease. In sum, our results indicate consistently an association between heart failure and decoupling of the precuneus from other brain regions being associated with social and executive functions. Further longitudinal studies are warranted elucidating etiopathological mechanisms.

## Introduction

Aging increases the risk to develop neurodegenerative diseases, most frequently Alzheimer’s disease^1^. Understanding pathophysiological mechanisms and developing strategies to prevent disease and modify its course are of uttermost importance. Recently, Walker et al.^2^ investigated plasma proteomic changes preceding the onset of dementia in a proteome-wide association study. Out of 4,877 plasma proteins in non-demented older adults in the Atherosclerosis Risk in Communities cohort, 83 proteins were associated with development of dementia over five years. Sixteen were also associated with late-life dementia risk if collected from plasma during mid-life, nearly 20 years earlier. Remarkably, one of these proteins was N-terminal prohormone of brain natriuretic peptide (NT-proBNP), an indicator of heart failure (HF). The prohormone NT-proBNP not only predicted development of dementia, but was even strongly associated with smaller brain volume in regions affected by Alzheimer’s disease. The authors suggested that—based on these associations—NT-proBNP beside other proteins should be considered as a potential therapeutic target. Based on these findings, studies are imperative exploring the consequences of HF on brain structure and function.

In general, HF reducing blood flow leads to insufficient oxygen supply throughout the organism including the brain^3^. Recently, we had investigated consequences of HF on brain structure in eighty patients with suspected coronary artery disease (CAD) at the Leipzig Heart Center in Germany in a case–control study^4^. Brain structure was assessed using gray matter density (GMD) measured with structural magnetic resonance imaging (MRI). Comparing patients with and without HF, GMD was diminished in patients with HF across various brain regions including the frontomedian cortex, parietal cortex, posterior cingulate cortex, and precuneus. The impact of HF on brain structure was further supported by biomarkers of HF. Cardiac left-ventricular ejection fraction (LVEF), expressing the percentage of blood pumped out with each contraction, correlated positively with GMD across the whole frontal and parietal medial cortex, whereas NT-proBNP was negatively associated with GMD in the medial and posterior cingulate cortex, hippocampus, and precuneus. Remarkably, these brain regions have been identified meta-analytically as core hubs involved in Alzheimer’s disease, the most frequent dementia syndrome, and its risk state mild cognitive impairment^1^. This meta-analysis including 2448 subjects revealed structural changes, i.e., atrophy, in the (trans-)entorhinal cortex/hippocampus, and functional alterations, here hypoperfusion and glucose hypometabolism, in the posterior cingulate cortex and precuneus.

In sum, our former results confirmed structural brain damage due to HF in areas essential for cognitive functions. However, decisive questions remain. Beside the impact on brain structure, one might assume that HF affects (even earlier) brain function. Hence, what about HF’s impact on functional parameters such as functional brain connectivity? Secondly, what are the neural correlates of low cognitive function? Thirdly, does HF have an impact on cognitive performance in the long-term? To shed more light onto these questions, we analyzed *functional* MRI data in exactly the same cohort that was previously studied with structural MRI^4^. We identified connectivity changes related to HF, revealed an association of this parameter with cognitive function, and show cognitive impairment in the long-term due to HF.

## Results

### Network centrality analysis: heart failure disconnects the precuneus from other brain regions

**Figure 1:**
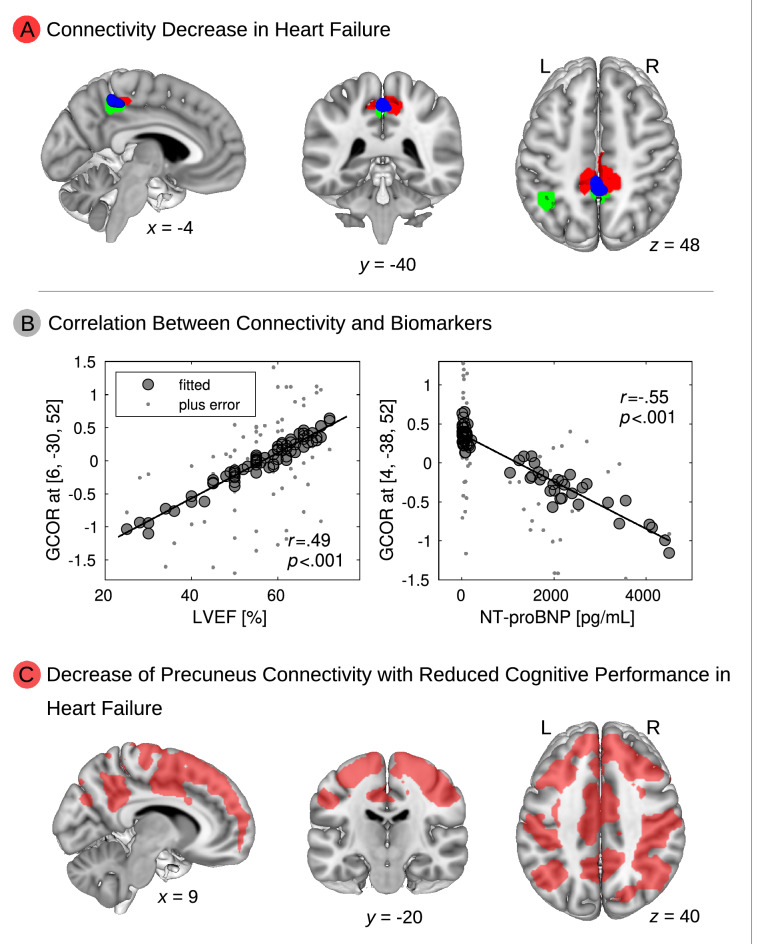
**Heart failure (HF) decreases brain connectivity in association with biomarker changes and cognitive decline.** (**A**) Connectivity is decreased in heart failure (HF) in the precuneus using global correlation (GCOR) as a centrality measure (red color). Results were obtained using a two-sample *t*-test between patients with (*N* = 33) and without HF (*N* = 42). The precuneus was also obtained when predicting conversion from the risk-state mild cognitive impairment to Alzheimer’s dementia (green color; Schroeter et al.^1^). The overlap between the connectivity finding and the meta-analysis is shown in blue color. (**B**) Across all patients (*N* = 75), a significant correlation was obtained between GCOR and HF-related biomarkers, i.e., left ventricular ejection fraction (LVEF), and N-terminal prohormone of brain natriuretic peptide (NT-proBNP) in the precuneus. Lower LVEF and higher NT-proBNP—both indicating HF—were associated with decreased GCOR. (**C**) Using the precuneus in a seed-based correlation analysis, decreased precuneus connectivity was found with lower cognitive performance in HF. In particular, an interaction between the factors HF and cognitive performance was obtained showing a significant group difference (HF vs. no-HF) with respect to decreased precuneus connectivity associated with lower cognitive performance in HF (red color). All analyses (**A, B, C**) were performed using age, sex, and body mass index as nuisance covariates. Significant results in the voxel-wise analyses were obtained with nonparametric permutation analysis using 10,000 permutations and threshold-free cluster enhancement (TFCE) with family-wise error correction (*p* < 0.05). x, y, z—coordinates of the Montreal Neurological Institute (MNI) stereotactic space. L—left, R—right.

Group comparison of brain network centrality between patients with (*N* = 33) and without HF (*N* = 42) showed a significant difference in precuneus connectivity (Fig. 1A, top panel, red color). HF was associated with decreased centrality in the precuneus. This regional centrality decrease was replicated for the comparison within patients with proven CAD, i.e., CAD patients with HF vs. without HF. Interestingly, the same group difference (HF vs. no-HF) was obtained with the threshold-free cluster enhancement (TFCE)^5^ technique and the LISA^6^ approach which are both based on nonparametric permutation analysis (see Supplementary Figure S1A, top panel, red color).

A further analysis investigated the correlation between brain connectivity and HF biomarkers across all participants. This analysis revealed a significant correlation between brain network centrality and the HF-related biomarkers LVEF and NT-proBNP (see Fig. 1B, middle panel). Lower LVEF was associated with decreased network centrality in the same region as obtained with the group analysis, namely the precuneus. Correspondingly, higher NT-proBNP concentrations were related to decreased centrality in this brain region. Note that NT-proBNP levels were not associated with the several cognitive parameters.

### Seed-based correlation analysis: dorsofrontoparietal regions mediate between heart failure and cognition

Furthermore, we investigated a potential relationship between precuneus connectivity and cognitive performance using a seed-based correlation analysis with the precuneus as seed-region. Comparing HF patients with high and low cognitive performance (i.e., above or below mean), we obtained a significant connectivity difference between precuneus and widely distributed cortical regions of the entire brain. Moreover, we obtained a significant interaction between the factors HF (HF vs. no-HF) and cognitive performance (high vs. low), i.e., diminished precuneus connectivity was associated with low cognitive performance in patients with HF but not in patients without HF (Fig. 1C, bottom panel, red color). Thus, this result reflects a significant decrease of connectivity between the precuneus and widely distributed cortical brain regions specifically within the group of patients with HF and low cognitive performance. Interestingly, this result was also obtained with both the TFCE^5^ technique and the LISA^6^ approach (see Supplementary Figure S1B, bottom panel, red color).

### Seed-based correlation analysis: dorsofrontoparietal regions are mainly related to executive functions, and to a lesser extent to memory and attention

**Figure 2:**
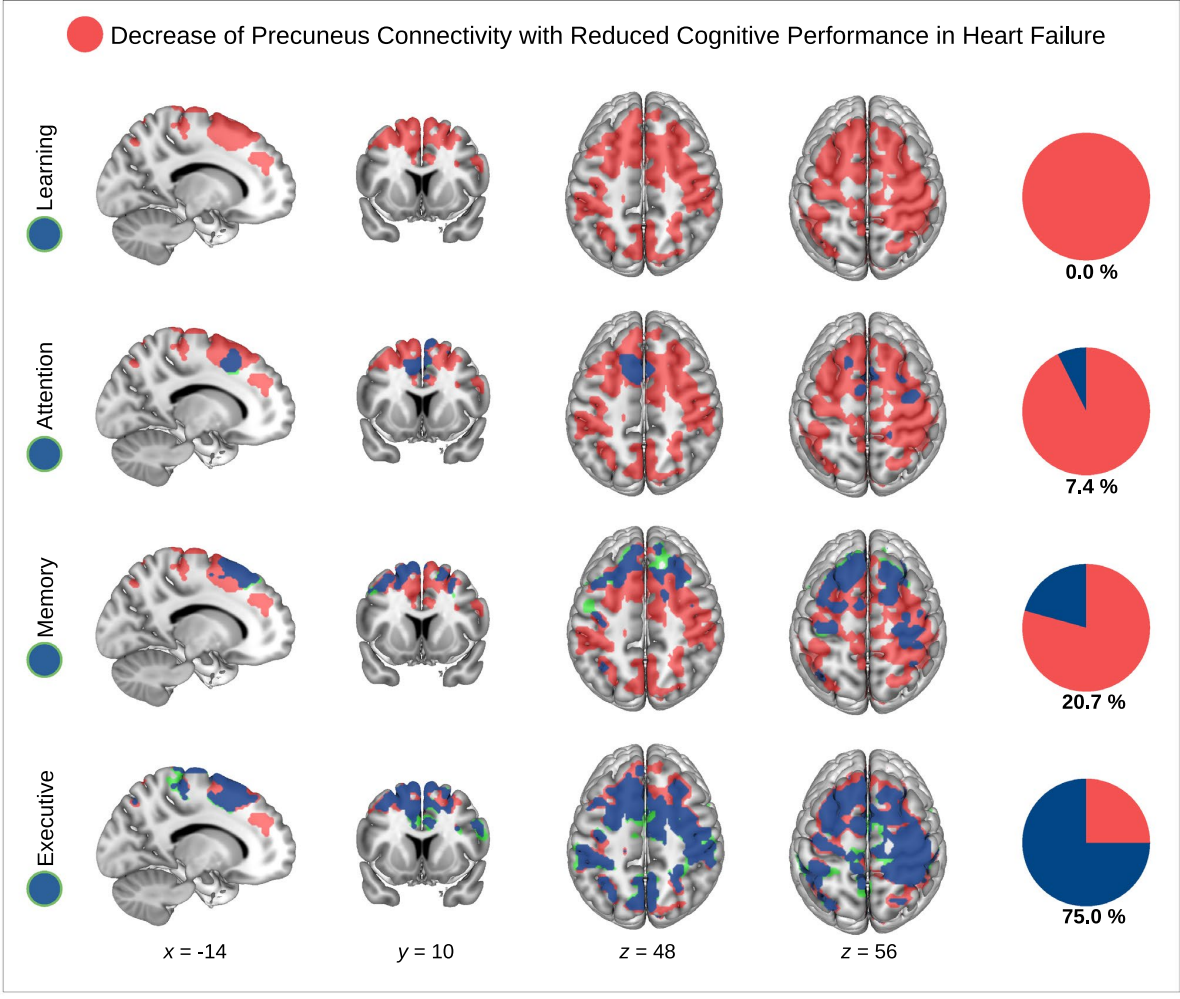
**Decreased precuneus connectivity with lower cognitive performance in heart failure (HF) is mainly related to executive functions, and, to a lesser extent, to memory and attention.** Using the precuneus in a seed-based correlation analysis across all HF patients, decreased precuneus connectivity was found with lower cognitive performance for the general score across all cognitive domains. In particular, an interaction between the factors HF and cognitive performance was obtained showing a significant group difference (HF vs. no-HF) with respect to decreased precuneus connectivity with lower cognitive performance for the general score across all cognitive domains (red color). In addition to the general score across all cognitive domains, the same analysis was repeated for each of the single cognitive domains, i.e., executive function, attention, memory and learning (green and blue color, overlap with the general score in blue color). Analysis was conducted across all available 75 patients. Pie charts on the right illustrate overlap between neural network for the general cognition score and respective specific cognitive domains (overlap again blue). All analyses were performed using age, sex, body mass index, and precuneus gray matter density as nuisance covariates. Significant results in the voxel-wise analyses were obtained with nonparametric permutation analysis using 10,000 permutations and threshold-free cluster enhancement (TFCE) with family-wise error correction (*p* < 0.05). *x, y, z*—coordinates of the Montreal Neurological Institute (MNI) stereotactic space. L—left, R—right.

To further elucidate the association between HF, precuneus connectivity, and cognition, we conducted additional analyses to deep-phenotype this relationship. In contrast to the categorial design described in the previous section, we used a factorial design (HF, cognitive performance) using the continuous values of cognitive performance (instead of using categories of high vs. low cognitive performance). Here, we again found a significant interaction between HF and cognitive performance reflecting a correlation between precuneus connectivity and cognitive performance in HF (Fig. 2, red color). In addition of using the general score across all cognitive domains, this analysis was repeated for each of the single cognitive domains, i.e., executive function, attention, memory and learning. Results are illustrated in Fig. 2. In HF patients, the relationship between precuneus connectivity and cognitive performance was mainly driven by executive function scores (75% overlap with general cognition result, see Fig. 2, bottom panel, blue color), whereas memory and attention did show weaker associations (20.7 and 7.4% overlap, respectively), and learning no association.

### Meta-analytical analysis: executive and social abilities are associated with neural networks affected by heart failure

In addition to our voxel-wise analyses, we validated the association between HF, brain connectivity, and cognition—as observed in our study—with meta-analytical techniques. We applied the so-called meta-analytical reading of symptoms (MARS) method^7^. This data-driven meta-analytical approach extracts specific cognitive functions associated with neural networks based on a large independent database with functional imaging data of more than 100,000 subjects. As region of interest, we used the precuneus resulting from the group comparison between HF and no-HF, and the brain regions mediating between HF, connectivity and *general* cognition to avoid circular approaches.

**Figure 3:**
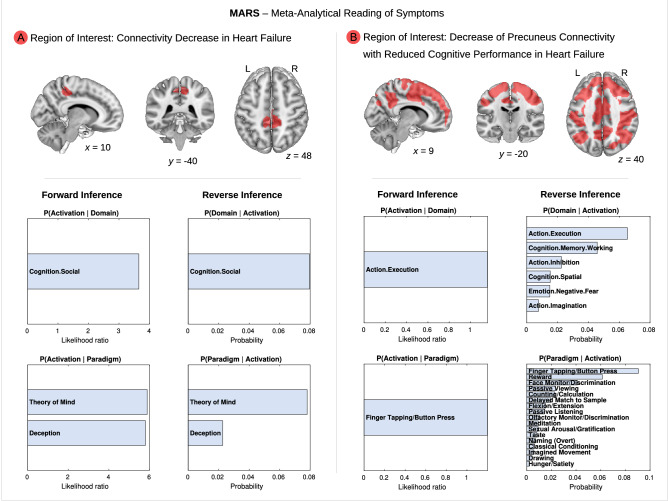
**Meta-analytical data-mining shows that neural networks affected by heart failure (HF) are mainly related to executive and social abilities—BrainMap database.** Top panel illustrates in red color (**A**) connectivity decrease in HF in the precuneus (left) and (**B**) the neural network originating from a seed-based correlation analysis showing decreased precuneus connectivity with reduced cognitive performance in HF (right; data are identical to Fig. 1A, C). These brain areas were used as regions of interest to extract related cognitive domains with meta-analytical reading of symptoms (MARS). Lower blue panels show results for forward (likelihood ratio) and reverse inference (probability), i.e., relevant associated cognitive domains and paradigms based on a very large independent cohort from the BrainMap database. x, y, z—coordinates of the Montreal Neurological Institute (MNI) stereotactic space. L—left, R—right.

Results are illustrated in Fig. 3. Upper panel shows in red respective regions of interest. We report results for both approaches of forward and reverse inference. The analysis extracted ‘cognition—social cognition’ as cognitive domain and ‘theory of mind’ and ‘deception’ as paradigm classes related to the precuneus shown to be disconnected due to HF in the group comparison between patients with HF vs. without HF (Fig. 3A, left).

A further analysis was conducted for the dorsofrontoparietal network revealed by an association analysis between connectivity, HF and general cognitive performance (see Fig. 3B, right). Here, the meta-analytic MARS approach revealed more complex results with ‘action execution’ as cognitive domain for both approaches of forward and reverse inference. The reverse inference approach indicated ‘cognition working memory’, ‘action inhibition’, ‘cognition spatial’, ‘emotion negative fear’ and ‘action imagination’ as further relevant domains. Both approaches identified ‘finger tapping/button press’ as relevant paradigm. The reverse inference approach extracted additionally more diverse paradigms (for details see Fig. 3B).

Taken together, the meta-analytical approach identified consistently social cognition as related to the precuneus, and executive functions as related to the dorsofrontoparietal region of interest. To further validate this finding, we conducted a conjunction analysis by overlapping the aforementioned result for the precuneus resulting from the group comparison HF vs. no-HF, and the dorsofrontoparietal network reflecting the interplay between brain connectivity, HF and general cognition with the correlates of social and executive functions in the Neurosynth^8^ database (https://neurosynth.org/). This database contains functional imaging data from a large independent cohort.

The meta-analysis was conducted for the term ‘social’ (1302 studies identified; 47,083 activations reported) and ‘executive’ (786; 28,937), because related terms contained a lower number of studies (social cognition 220, theory mind 181; executive control 230, executive function 154, executive functions 128). Finally, we conducted a conjunction analysis between the neural correlates of the meta-analysis on social cognition and executive functions from Neurosynth^8^, and the precuneus and dorsofrontoparietal regions of interest, respectively.

**Figure 4:**
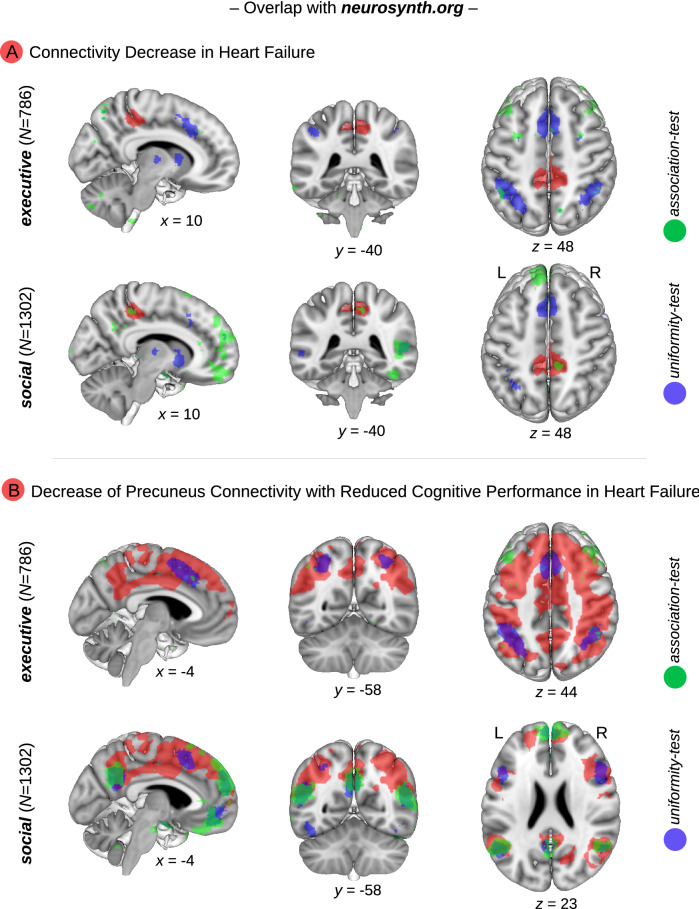
**Meta-analytical data-mining shows that neural networks affected by heart failure (HF) are mainly related to executive and social abilities—Neurosynth database.** Brain connectivity decrease in HF in the precuneus (top panel, **A**) and the neural network originating from a seed-based correlation analysis showing decreased precuneus connectivity with reduced cognitive performance in HF (bottom panel, **B**) are illustrated in red color (data are identical to Fig. 1A, C). Overlap between the aforementioned regions and neural correlates of social and executive functions as extracted from the meta-analytical Neurosynth database, another very large independent cohort. Results are shown for the association test (green color) and the uniformity test (blue color). False discovery rate of 0.01. x, y, z—coordinates of the Montreal Neurological Institute (MNI) stereotactic space. L—left, R—right.

Results are illustrated in Fig. 4. We detected a small overlap between the neural correlates of the meta-analysis on social functions in the Neurosynth database and the precuneus disconnected by HF, whereas the precuneus analysis revealed no overlap with executive functions as expected (Fig. 4A, upper panel). Furthermore, we detected a large regional overlap between executive and social functions in the Neurosynth database and brain regions associated with precuneus connectivity, HF and general cognitive performance in our study (Fig. 4B, bottom panel). This finding underlines the importance of social and executive (dys)function in HF.

### Follow-up investigation: heart failure is associated with higher amount of cognitive impairment in the long-term

As revealed by the cognitive assessment, 15.2% of patients with HF and 19.0% of patients without HF showed mild cognitive impairment, equivalent to mild neurocognitive disorder according to DSM-5 during first assessment, without any subject fulfilling criteria for dementia, equivalent to major neurocognitive disorder (*p* > 0.05 in Fisher's exact test; for details see Table 1). To assess the course of cognitive performance in the long-term, we reinvestigated study participants after 8.8 ± 0.3 years. Of the 80 patients included in the initial study^4^, 31 patients (13 with HF, 18 without HF) could be re-investigated. Of the other participants, four had deceased in the meantime, 19 had no interest in reinvestigation, and 26 could not be contacted (49 participants without follow-up investigation).

**Table 1:** **Demographic and clinical data of the studied subgroups.**

	HF	no-HF	*P*
Group size	33	42	
Females/males^†^	8/25	12/30	0.79
Age^**+**^, years	53.8 ± 5.8	55.9 ± 5.0	0.10
BMI^**+**^, kg/m^2^	28.7 ± 4.2	28.1 ± 4.0	0.53
Smoking^†^, never/former/present	7/18/8	13/17/12	0.50
Diabetes^†^, yes/no	8/25	9/33	0.79
Hypertension^†^, yes/no	18/15	31/11	0.09
RR syst^**+**^, mm Hg	132.6 ± 16.8	135.4 ± 14.5	0.44
RR dia^**+**^, mm Hg	82.4 ± 8.3	82.7 ± 8.5	0.90
Mild/major NCD/normal Cog^†^	5/0/28	8/0/34	0.76
Mild/major NCD/Normal Cog^†^—follow-up*	6/3/4	5/5/8	0.65
Fazekas score^†^, 0/1/2/3	11/19/3/0	16/20/5/1	0.86
Troponin T^**+**^, pg/ml	2474.2 ± 1992.6	6.6 ± 6.3	< 0.001
CK^**+**^, µkat/l	31.61 ± 25.56	2.09 ± 1.30	< 0.001
CK-MB^**+**^, µkat/l	3.77 ± 2.76	0.25 ± 0.06	< 0.001
LVEF^**+**^	48.1 ± 11.0	63.1 ± 5.4	< 0.001
NT-proBNP^**+**^, pg/ml	2668.0 ± 1574.5	67.8 ± 46.9	< 0.001

HF: heart failure; no-HF: group patients without heart failure; BMI: body mass index; RR syst: systolic blood pressure; RR dia: diastolic blood pressure; Mild/Major NCD: mild/major neurocognitive disorder; Normal Cog: cognitively normal; Troponin T: high sensitive troponin T; CK: creatine kinase; CK-MB: CK isoenzyme muscle brain; LVEF: left ventricular ejection fraction; NT-proBNP: N-terminal prohormone of brain natriuretic peptide; ^**+**^two-sample *t*-test, two-sided, unequal variance; ^†^Fisher’s exact test, two-sided. *Note that the follow-up investigation after approximately nine years could re-investigate the original cohort only partly.

The cognitive follow-up assessment revealed higher rates of impairment in participants with HF in comparison to participants without HF. After approximately nine years, 46.2% of the participants with HF fulfilled criteria for mild, and 23.1% for major neurocognitive disorder, but only 27.8% for mild or major neurocognitive disorder, respectively, of the participants without HF (*p* > 0.05; Fisher’s exact test). Accordingly, HF was associated with cognitive impairment in 69.3% of the cases after approximately nine years, in contrast to 55.6% in the no-HF group. Of note, the distribution between impaired cognitive domains differed between both groups. HF was characterized by main impairment in the social domain (38.5% of all re-investigated subjects) followed by the perceptual motor domain (30.8%; further domains language 23.1%, attention 23.1%, memory 15.4%, executive 0%). For the cohort without HF, deficits in the perceptual motor domain (27.8%), followed by memory (22.2%), dominated (further domains social cognition 16.7%, attention 11.1%, executive 11.1%, language 0%).

## Discussion

Our study aimed at answering several research questions. Firstly, one can conclude that HF not only has an important impact on brain structure as evaluated by gray matter changes^4^ but also on brain function as assessed by functional MRI during resting state. Here, we identify the precuneus as the decisive brain connectivity hub that has been decoupled from other brain regions due to HF. This brain region is related to several cognitive functions as visuo-spatial imagery, episodic memory retrieval, and self-processing function, such as first-person perspective taking and experience of agency^9^, and in addition, the precuneus is one of the brain structures with highest resting metabolic rates^9^. Secondly, our study identifies the neural correlates of HF-related low cognitive function. Investigating the relationship between precuneus connectivity and cognitive performance, we observed an HF-related precuneus dysconnectivity associated with lower cognitive performance. In particular, patients with HF and lower cognitive performance showed a reduced brain connectivity between the precuneus and widespread cortical regions that is in line with current neuroanatomical considerations^9^. Supporting the decisive role of the precuneus for cognitive deficits in HF, Horstmann et al.^10^ have shown that cardiac arrest diminishes gray matter volume of the precuneus in correlation with memory decline.

As mentioned in the introduction, Walker et al.^2^ identified NT-proBNP—collected during mid-life—as a predictor for the development of late-life dementia approximately 20 years later. They demonstrated an association of this HF biomarker with brain atrophy regionally specific for Alzheimer’s disease. We conducted a conjunction analysis to further investigate the possible association between the regions affected by HF in our study and the regions indicating development of Alzheimer’s dementia as reported in the study by Schroeter et al.^1^ This conjunction analysis identified the brain regions overlapping for both analyses. Interestingly, the same region, i.e., the precuneus, was identified in the aforementioned comprehensive quantitative meta-analysis^1^ when investigating converters and non-converters from the risk state mild cognitive impairment to Alzheimer’s dementia, here predicting conversion in approximately two years (Fig. 1, upper panel, green color, overlap with centrality difference in blue). Our conjunction analysis between brain connectivity changes in HF and neural correlates predicting conversion from mild cognitive impairment to Alzheimer’s disease may suggest a higher vulnerability to develop neurodegenerative cognitive decline with HF.

This hypothesis might be supported by studies showing Alzheimer’s disease-related histopathological changes that occur already in midlife, approximately 20 years before symptom onset in genetic, autosomal dominant and in much more frequent sporadic Alzheimer’s disease^11–16^. Furthermore, our results neatly fit into the vascular hypothesis framework of Alzheimer’s disease that assumes vascular dysfunction as a crucial factor in its pathogenesis^17–19^. Here, changes in cerebral blood flow are assumed to be present even before other pathology of Alzheimer’s disease, such as beta-amyloid accumulation, brain atrophy or clinical symptoms. Indeed, studies confirm hypoperfusion in parietal areas, including the precuneus/posterior cingulate cortex, co-localized with regional glucose metabolism indicating neuronal dysfunction not only in Alzheimer’s disease, but also its risk-state mild cognitive impairment^1^. Other studies demonstrate that changes in cerebral blood flow predict conversion from this risk state to Alzheimer’s dementia^18,19^. New concepts even connect HF and Alzheimer’s disease in framing the heart-brain axis as an organ system with similar pathogenic mechanisms requiring new interdisciplinary stances^20^.

The association between HF and dementia has to be proven in longitudinal studies involving also specific biomarkers of Alzheimer’s disease such as amyloid, tau or phospho tau from positron emission tomography, cerebrospinal fluid or serum/plasma. Furthermore, neurofilaments might be of interest, and the association of these biomarkers with brain connectivity in cohorts with HF.

To further elucidate the relationship between HF and cognitive function, we conducted in-depth phenotyping analyses. Here, we found that the dorsofrontoparietal network mediating between HF, connectivity to the precuneus, and general cognitive performance, was mainly related to executive functions and to a lesser extent to memory and attention. Note that social cognition was not included as a cognitive domain in this neurocognitive battery. Furthermore, our meta-analytical data-mining approaches supported the assumption that this neural network is mainly related to executive functions, as reflected in the data-driven MARS approach^7^ with dominating results for action execution, cognition working memory, action inhibition, and action imagination, beside other more widespread cognitive functions in the paradigm approach. Notably, applying the MARS approach to the precuneus identified in the HF vs. no-HF comparison social cognition and theory of mind as dominating cognitive domains. These results were supported by an independent meta-analytical approach (Neurosynth^8^ database) with a conjunction between the two regions of interest and social and executive networks, where the frontoparietal connectivity showed additionally an overlap with networks for social cognition.

The relevance of our findings was further validated by the follow-up investigation in our cohort after approximately nine years to answer questions about potential conversion to mild or major neurocognitive disorder (equivalent to mild cognitive impairment or dementia) and the pattern of cognitive deficits in the long-term. Such longitudinal studies are crucial to prove our hypothesis as our study investigated mid-term effects only. In agreement with our hypothesis, participants with HF showed higher prevalence of cognitive impairment than participants without HF. Surprisingly, social cognitive deficits were the dominant cognitive domain impaired by HF. This result fits very well with our meta-analytical results, extracting this domain as dominant for the precuneus in the baseline investigation.

Our findings answer the third research question as raised in the introduction. They support the hypothesis that HF leads to cognitive impairment in the long-term, but contradict a direct association with Alzheimer’s disease etiology as memory dysfunction—typical in this disease^1^—did not dominate in our cohort. Moreover, one has to mention here that regions altered in Alzheimer’s disease go far beyond alterations in the precuneus and involve other etiological mechanisms, and that, on the other hand, precuneus connectivity might be influenced by several factors. On the other hand, social cognition has to be regarded as under-investigated in Alzheimer’s disease^21^, and HF hampering final conclusions.

Our long-term follow-up yields promising pilot results, although we could reinvestigate less than 50% of our original cohort. Executive deficits were not found in the follow-up in HF, at least to an extent of mild or major neurocognitive impairment. This result might be due to our tight definition of the executive domain in the follow-up investigation as it was based only on the trail making test B/A ratio. In contrast, the cognitive baseline examination assessed executive functions with several tests/paradigms, which was also the case in the meta-analytical datasets. Future studies shall transcend these limitations by including more subjects and consistent neuropsychological test batteries.

Interestingly, our findings are in agreement with a systematic review^22^ indicating that HF increases the risk for cognitive decline. On the other hand—turning the focus of attention to plasticity—this review shows further that interventions improving cardiac function are able to stabilize or even improve cognitive function in this patient cohort. We believe that this perspective, underlined by systematic and quantitative meta-analyses in the future, will open the horizon to developing transdisciplinary intervention strategies.

Finally, we want to discuss potential limitations of our study and suggest new perspectives following our study. Overall, a higher rate of patients in the HF group (34%) than the no-HF group (16%) had to be excluded as they missed the date of examination, data had insufficient quality, for instance due to technical problems or movement artifacts, or anatomical MRI did show ischemic brain lesions potentially interfering with brain connectivity. Although this imbalance might have confounded our results, higher exclusions rates had to be expected in the HF group. As already discussed, we could also investigate a part of the participants during our long-term follow-up only.

Moreover, it is well known that aging has a decisive impact on cognitive performance. Here, we used values for neuropsychological tests that had already been normalized according to age, and furthermore to education and sex, if available. Functional MRI, LVEF, and NT-proBNP levels might be influenced by factors such as aging; NT-proBNP levels furthermore by obesity and kidney disease. Using resting state functional MRI, Klaassens et al.^23^ have shown reduced functional brain connectivity with both, normal aging and Alzheimer’s disease. Whereas normal aging led to widespread decreases in brain connectivity, Alzheimer’s disease affected connectivity between the default mode network and precuneus only. In agreement, another study with functional near infrared spectroscopy imaging^24^ has shown a decline in spontaneous low-frequency oscillations in the aging brain—oscillations that are contained in our frequency band of functional MRI.

Regarding age and obesity, statistical analyses did not show any differences for age, sex and body mass index between groups in our study (see Table 1). Furthermore, all statistical analyses were performed using age, sex, and body mass index as nuisance covariates. Hence, we controlled for these factors excluding a potential bias here. For kidney disease, we had no explicit information, such as creatinine levels, which might be regarded as a limitation. Moreover, vascular risk factors, their consequences and cardiovascular medications might have an impact on cerebral blood flow. Of note, frequency of diabetes mellitus, smoking, obesity, arterial hypertension, and small vessel disease (Fazekas score) did not differ between patients with and without HF (Table 1). Note that we controlled explicitly for stroke in structural MRI data for each subject, and excluded two subjects from the HF group due to ischemic lesions potentially interfering with connectivity measures. Of course, cardiovascular medications might generally have an impact on cerebral blood flow. Based on vascular risk factors one might assume that medication for risk factors mainly was comparable between groups. However, systematic information for such medication for each participant was not available. Although this has to be regarded as a limitation, we are confident that such drugs would not have a relevant impact on results, because we normalized data. Even if cerebral blood flow would be influenced, i.e., presumably diminished, one would assume here general effects across the whole brain and not region-specific effects. Future studies are warranted to systematically investigate possible effects of cardiovascular medications on brain connectivity.

## Conclusion

In sum, we investigated the impact of heart failure on brain connectivity using functional magnetic resonance imaging at resting state. Our study shows brain connectivity alterations related to heart failure and cognitive performance. Heart failure decreases brain connectivity in the precuneus. Precuneus dysconnectivity was related to biomarkers of heart failure, i.e., left ventricular ejection fraction and N-terminal prohormone of brain natriuretic peptide, and low cognitive performance, mainly executive function. Meta-analytical data-mining approaches revealed that social and executive cognitive functions are mainly associated with those neural networks. Notably, the precuneus, as identified in our study in a mid-life cohort, represents one of the central functional hubs affected by Alzheimer’s disease. A long-term follow-up investigation in our cohort after approximately nine years revealed more severe cognitive impairment in the group with heart failure than controls, where social cognition was the cognitive domain mainly affected, and not memory such as in Alzheimer’s disease. In sum, our results indicate an association between heart failure and decoupling of the precuneus from other brain regions being associated with social and executive functions. Further longitudinal studies are warranted elucidating etiopathological mechanisms.

## Methods

### Patient cohort

As described in Mueller et al.^4^, the same cohort of 80 patients (22 females; age, 54.9 ± 5.3 years; mean ± standard deviation) initially presenting with clinical symptoms of potential CAD—such as chest pain and shortness of breath—and leading to hospital admission participated in the study. Patients’ recruitment, selection, demographic and clinical characteristics are described in detail^4^. Study participants took part in the Leipzig Heart Study (total cohort *N* = 2884 patients with suspected CAD; initial visit within a time window of 5.5 years between December 2006 and July 2012). Patients were recruited to participate in this study within one year at the time of a follow-up measurement with treated and, accordingly improved, HF (between September 2012 and September 2013). Cognitive tests and MRI were performed 3.5 ± 1.3 years after the initial visit to assess mid-term changes.

Patient selection was performed according to the guidelines of the European Society of Cardiology^25^ based on NT-proBNP serum concentration obtained in acute setting. For patients with an intermediate age, HF is likely to be associated with NT-proBNP concentrations above 900 pg/mL (see Table 2 in Mueller et al.^26^). Based on this definition, the study included 50 patients with HF (*HF* group) and 50 matched patients without HF (*no-HF* group). Please, refer for workflow for patient selection to online Fig. 1 in the supplement of Mueller et al.^4^ Matching criteria included age, sex, diabetes mellitus, smoking status, carotid intima media thickness and carotid plaque presence. Note that 15 patients with HF and five patients without HF had to be excluded as they missed the date of examination or data had insufficient quality such as due to technical problems or movement artifacts. In contrast to our preceding work^4^, we further excluded two patients with HF due to ischemic brain lesions and three patients without HF showing acute NT-proBNP levels below 900 pg/mL but above 300 pg/mL (also denoted as “gray zone” between HF and no-HF in Mueller et al.^26^). Thus, the remaining group size was 33 patients in the HF group and 42 patients in the no-HF group. Accordingly, 75 patients were included, leading to an overall exclusion rate of 34% in the HF group and 16% of the no-HF group. For demographic and clinical characteristics of the studied groups, please refer to Table 1.

### Biomarker assessment

In all patients, investigations included several blood parameters implying NT-proBNP, echocardiography with determination of LVEF, and cardiac catheterization beside thoroughly clinical examination. Thus, both LVEF and NT-proBNP were obtained in an acute state right after hospitalization. LVEF was measured by biplane 2-dimensional measurements for end-diastolic (EDV) and end-systolic (ESV) left ventricular volumes: LVEF = (EDV-ESV)/EDV. NT-proBNP levels were analyzed with Roche Modular analyzer (Roche Diagnostics, Germany). Biomarker assessment proved CAD in all HF patients. Moreover, CAD was also shown in 20 patients of the no-HF group.

Note that statistical analyses did not reveal any differences for sex, age, obesity, vascular risk factors diabetes mellitus, smoking, obesity (body mass index), and arterial hypertension between the subgroups HF and no-HF (Table 1).

### Neuropsychological assessment

Cognitive performance was assessed with a comprehensive test battery covering the cognitive domains attention (trail making test A^27,28^, and test battery of attentional processes^29^), executive function (trail making test B^27,28^, hamasch 5-point test^30^ revised, Regensburg word fluency test^31^, standardized Link’s probe^32^, Stroop test^33,34^), learning and memory (California verbal learning test^35^, and Rey-Osterrieth complex figure test^36^) at the time of the MRI acquisition (for more information see Mueller et al.^4^). Individual raw values were transformed into age- and (if available) sex-matched normalized values according to the Diagnostic and Statistical Manual of Mental Disorders, 5th edition, DSM-5^37^. Mean averages were calculated for all cognitive domains, and the average of all single domain values was used as a marker of global *cognitive performance*, which was used in subsequent brain connectivity analysis. To further elucidate the relationship between brain connectivity, HF and cognition, we added single domain analyses of cognitive performance for deep-phenotyping, i.e., separately for executive function, attention, memory, and learning.

Five of the 33 patients with HF (15.2%) and eight of the 42 patients without HF (19.0%) showed mild cognitive impairment or mild neurocognitive disorder according to DSM-5, without significant differences between groups (*p* > 0.05 in Fisher's exact test). None of the patients fulfilled any criteria for dementia or major neurocognitive disorder.

### Image acquisition and analysis

Functional MRI was acquired covering the whole brain with a 3-T MAGNETOM Verio scanner (Siemens Healthineers, Erlangen, Germany) and a 32-channel head receive array with a T2*-weighted gradient-echo echo-planar imaging (EPI) sequence (repetition time 2 s; echo time 30 ms; flip angle 90°; pixel bandwidth 1953 Hz). Functional images were acquired with the following image dimensions: acquisition matrix 64 × 64 pixels, 30 axial slices with a slice thickness of 4 mm (0.8 mm gap, ascending slice order), nominal image resolution 3 × 3 × 4.8 mm^3^. For each participant, 300 functional volumes were acquired resulting in a total scanning duration of 10 min. Scanning was performed in resting state, i.e., participants were asked to focus a fixation cross without carrying out any specific cognitive task.

Image preprocessing, nuisance regression, and connectivity analyses were performed using the conn-toolbox rev 21a^38^ and SPM12 rev. 7771^39^ (Wellcome Centre for Human Neuroimaging, University College London, UK) with Matlab 9.12 rev 2022a (The MathWorks Inc., Natick, MA). For each participant, an individual brain connectivity map was generated using network centrality (global correlation, GCOR) reflecting the hubness of each voxel of the brain. Subsequent group and correlation analyses were processed across all GCOR images using a general linear model implementing a two-sample *t*-test to detect GCOR differences between patients with and without HF. Correlation analyses included either individual LVEF or NT-proBNP values as a covariate of interest, and all models included age, sex, and body mass index (BMI) as covariates of no interest (nuisance covariates). Analyses were performed at the whole-brain level, i.e., for all voxels within the brain.

Further analysis aimed at seed-based connectivity: For each patient, a seed-based correlation map was generated using the precuneus as seed-region. Subsequent group analysis was performed using a flexible factorial 2 × 2 design with the factors HF (HF vs. no-HF) and cognitive performance (high vs. low) in order to investigate a potential interaction between both factors. In particular, we checked for a group difference (HF vs. no-HF) showing diminished precuneus connectivity with lower cognitive performance in HF but not in no-HF.

In addition to the 2 × 2 factorial design (described above), another factorial model was used with the same factors but implementing cognitive performance as continuous variable obtained from the neuropsychological assessment. Here, we again tested for a potential interaction between HF and cognitive performance. This analysis was performed using the global cognitive performance (using the average of cognitive performance across all cognitive domains) but also repeated for each single cognitive domain, i.e., for cognitive performance related to executive function, attention, memory, and learning. These analyses also included precuneus gray matter density obtained from a preceding analysis^4^ as covariate of no interest (in addition to age, sex, and BMI).

As nonparametric permutation test was found to produce nominal results for group inference based on functional MRI data^40^, we performed nonparametric permutation analysis using the TFCE^5^ technique with all group analyses. Significant differences were obtained with the TFCE toolbox rev. 254 (Structural Brain Mapping Group, University of Jena, Department of Neurology, Germany) using 10,000 permutations with *p* < 0.05 using family-wise error (FWE) correction. In addition, all group analyses were also performed using the LISA^6^ approach using 10,000 permutations with controlling the false discovery rate (FDR) using an alpha level of 0.05.

### Meta-analytical reading of symptoms (MARS) in the BrainMap database

Our imaging analysis based on functional MRI during resting state revealed brain networks associated with HF and with general cognitive performance. To extract the specific cognitive functions associated with these brain networks we applied data-driven meta-analytical approaches. Recently, we have introduced a method coined Meta-Analytical Reading of Symptoms (MARS)^7^. Beyond predicting symptoms in single subjects for personalized medicine, we have applied the same approach in meta-analytical studies to conceptualize neuropsychiatric diseases, here behavioral variant of frontotemporal dementia^41^.

For MARS, we calculated behavioral domain profiles for brain regions received in the connectivity analyses. Behavioral domain profiles extract the behavioral, psychological, or mental correlates related to a specific brain region^42^. The taxonomy includes five main behavioral domain categories, namely action, cognition, emotion, interoception, and perception with related subcategories and several paradigm classes representing specific tasks. This meta-analytic method is performed in a probabilistic functional brain atlas, the BrainMap database (http://www.brainmap.org/) containing whole brain functional imaging data from more than 100,000 subjects. For details, please refer to Lancaster et al.^42^ and Schroeter et al.^7,41^.

### Extracting neural networks for cognitive functions in the Neurosynth database

Moreover, we went to the Neurosynth^8^ database to validate associated networks, i.e., proving that networks detected in our analysis in HF, brain connectivity and cognition, are related to specific cognitive functions. Here, we conducted a meta-analysis across imaging studies from the literature with Neurosynth (http://www.neurosynth.org) to quantitatively extract the neural networks associated with social and executive functions. Neurosynth is a platform for large-scale, automated synthesis of functional MRI data including 507,891 activations from 14,371 studies (1st December 2022). As an automated brain-mapping framework, Neurosynth applies text-mining and meta-analytic techniques to generate a large database of mappings between neural and cognitive states.

Neurosynth^8^ automatically extracts activation coordinates and frequently used terms from published neuroimaging articles. To date, 1334 terms are contained in the database enabling meta-analyses. The entire database of coordinates is divided into two sets for each term of interest, these that are reported in articles containing the term, and those that are reported in articles not containing the term. Thereafter, the algorithm compares the coordinates reported for studies with and without the term of interest. Resulting images are corrected for multiple comparisons with a false discovery rate of 0.01. We included only positive results, and report results for the association and uniformity test (corresponding approximately to reverse and forward inference maps; for details see http://www.neurosynth.org). To guarantee high statistical power and validity, the meta-analysis was conducted for the term containing the highest number of studies, respectively. For methods applied, please refer also to other papers using the same approach^43–45^.

### Longitudinal neuropsychological follow-up assessment

To investigate how many participants developed a cognitive deterioration in the long-term, we re-invited participants and reassessed their cognitive performance. Because we laid the focus on dementia and its risk states, we applied well established dementia screening and social cognition tests for the follow-up study, i.e., the Consortium to Establish a Registry for Alzheimer's Disease (CERAD Plus battery)^46^, complemented by the Reading the Mind in the Eyes test (RMET)^47,48^. Herewith, we covered the cognitive domains suggested in the DSM-5^37^, i.e., attention (trail making test A^27,28^), executive function (trail making test B divided by version A^27,28^), learning and memory (word list immediate/delayed recall, recognition, and figures delayed recall), language (phonemic and verbal/semantic fluency, Boston naming test^49^), perceptual motor (figures copy) and social cognition (RMET)^47,48^. Individual raw values were transformed into age-, sex- (RMET) and additionally education-(CERAD Plus battery)-matched normalized values and mean averages were calculated for all cognitive domains as already described before.

### Ethics approval and patient and public involvement

The study was carried out in accordance with the Declaration of Helsinki and approved by the Ethics Committee of the University of Leipzig (ID 099-12-05032012). All participants signed written informed consent. Patients and the public were involved by briefing on study results, for instance via press releases, and discussing further study design, such as follow up investigations.

### Diversity statement

 Institutions involved in the study support diversity and consider it as a prerequisite for excellent science. Diversity is also mirrored in the group of authors involved in the study.

## Supplementary Information

Supplementary Legends.

Supplementary Figure 1.

## Data Availability

Datasets analyzed during the current study are available on reasonable request. All data will be anonymized. Functional MRI data will be available as smoothed preprocessed images in the NIfTI format without any personal meta-data.

## References

[1] Schroeter ML, Stein T, Maslowski N, Neumann J (2009). Neural correlates of Alzheimer's disease and mild cognitive impairment: a systematic and quantitative meta-analysis involving 1351 patients. Neuroimage.

[2] Walker KA (2021). Large-scale plasma proteomic analysis identifies proteins and pathways associated with dementia risk. Nat. Aging.

[3] Ovsenik A, Podbregar M, Fabjan A (2021). Cerebral blood flow impairment and cognitive decline in heart failure. Brain Behav..

[4] Mueller K (2020). Brain damage with heart failure: Cardiac biomarker alterations and gray matter decline. Circ. Res..

[5] Smith SM, Nichols TE (2009). Threshold-free cluster enhancement: addressing problems of smoothing, threshold dependence and localisation in cluster inference. Neuroimage.

[6] Lohmann G (2018). LISA improves statistical analysis for fMRI. Nat. Commun..

[7] Schroeter ML, Eickhoff SB, Engel A (2020). From correlational approaches to meta-analytical symptom reading in individual patients: Bilateral lesions in the inferior frontal junction specifically cause dysexecutive syndrome. Cortex.

[8] Yarkoni T, Poldrack RA, Nichols TE, Van Essen DC, Wager TD (2011). Large-scale automated synthesis of human functional neuroimaging data. Nat. Methods.

[9] Cavanna AE, Trimble MR (2006). The precuneus: a review of its functional anatomy and behavioural correlates. Brain.

[10] Horstmann A (2010). Resuscitating the heart but losing the brain: brain atrophy in the aftermath of cardiac arrest. Neurology.

[11] Bateman RJ (2012). Clinical and biomarker changes in dominantly inherited Alzheimer's disease. N. Engl. J. Med..

[12] Braak H, Del Tredici K (2012). Alzheimer's disease: pathogenesis and prevention. Alzheimer’s Dement..

[13] Braak H, Del Tredici K (2015). The preclinical phase of the pathological process underlying sporadic Alzheimer's disease. Brain.

[14] Jansen WJ (2022). Prevalence estimates of amyloid abnormality across the Alzheimer disease clinical spectrum. JAMA Neurol..

[15] Jansen WJ (2015). Prevalence of cerebral amyloid pathology in persons without dementia: A meta-analysis. JAMA.

[16] Villemagne VL (2013). Amyloid beta deposition, neurodegeneration, and cognitive decline in sporadic Alzheimer's disease: A prospective cohort study. Lancet Neurol..

[17] Apatiga-Perez R (2022). Neurovascular dysfunction and vascular amyloid accumulation as early events in Alzheimer's disease. Metab. Brain Dis..

[18] de la Torre J (2018). The vascular hypothesis of Alzheimer's disease: A key to preclinical prediction of dementia using neuroimaging. J. Alzheimer’s Dis..

[19] Hays CC, Zlatar ZZ, Wierenga CE (2016). The utility of cerebral blood flow as a biomarker of preclinical Alzheimer's disease. Cell Mol. Neurobiol..

[20] Daniele G, DiLucia S, Masci PG, Del Monte F (2020). Heart and brain: Complex relationships for left ventricular dysfunction. Curr. Cardiol. Rep..

[21] Roheger M (2022). Progression of socio-cognitive impairment from healthy aging to Alzheimer's dementia: A systematic review and meta-analysis. Neurosci Biobehav Rev.

[22] Hajduk AM, Kiefe CI, Person SD, Gore JG, Saczynski JS (2013). Cognitive change in heart failure: A systematic review. Circ. Cardiovasc. Qual. Outcomes.

[23] Klaassens BL (2017). Diminished posterior precuneus connectivity with the default mode network differentiates normal aging from Alzheimer's disease. Front. Aging Neurosci..

[24] Schroeter ML, Schmiedel O, von Cramon DY (2004). Spontaneous low-frequency oscillations decline in the aging brain. J. Cereb. Blood Flow Metab..

[25] McDonagh TA (2021). 2021 ESC Guidelines for the diagnosis and treatment of acute and chronic heart failure. Eur. Heart J..

[26] Mueller C (2019). Heart Failure Association of the European Society of Cardiology practical guidance on the use of natriuretic peptide concentrations. Eur. J. Heart Fail..

[27] Corrigan JD, Hinkeldey NS (1987). Relationships between parts A and B of the Trail Making Test. J. Clin. Psychol..

[28] Linari I, Juantorena GE, Ibanez A, Petroni A, Kamienkowski JE (2022). Unveiling Trail Making Test: Visual and manual trajectories indexing multiple executive processes. Sci. Rep..

[29] Zimmenmann, P. & Fimm, B. in *Applied Neuropsychology of Attention. Theory, Diagnosis and Rehabilitation* (eds M. Leclercq & P. Zimmermann) 110–151 (Psychology Press, 2002).

[30] Tucha L, Aschenbrenner S, Koerts J, Lange KW (2012). The five-point test: reliability, validity and normative data for children and adults. PLoS ONE.

[31] Aschenbrenner S, Tucha O, Lange KW (2001). Regensburger Wortflüssigkeits-Test.

[32] Metzler P (2012). Standardisierte Link’sche Probe.

[33] Stroop JR (1935). Studies of interference in serial verbal reactions. J. Exp. Psychol..

[34] Zysset S, Muller K, Lohmann G, von Cramon DY (2001). Color-word matching stroop task: Separating interference and response conflict. Neuroimage.

[35] Elwood RW (1995). The California Verbal Learning Test: Psychometric characteristics and clinical application. Neuropsychol. Rev..

[36] Shin MS, Park SY, Park SR, Seol SH, Kwon JS (2006). Clinical and empirical applications of the Rey-Osterrieth Complex Figure Test. Nat. Protoc..

[37] Association AP (2013). Diagnostic and Statistical Manual of Mental Disorders.

[38] Whitfield-Gabrieli S, Nieto-Castanon A (2012). Conn: a functional connectivity toolbox for correlated and anticorrelated brain networks. Brain Connect.

[39] Statistical Parametric Mapping (2007). The Analysis of Functional Brain Images.

[40] Eklund A, Nichols TE, Knutsson H (2016). Cluster failure: Why fMRI inferences for spatial extent have inflated false-positive rates. Proc. Natl. Acad. Sci. USA.

[41] Schroeter ML (2014). Conceptualizing neuropsychiatric diseases with multimodal data-driven meta-analyses: The case of behavioral variant frontotemporal dementia. Cortex.

[42] Lancaster JL (2012). Automated regional behavioral analysis for human brain images. Front. Neuroinform..

[43] Eslinger PJ (2021). The neuroscience of social feelings: Mechanisms of adaptive social functioning. Neurosci. Biobehav. Rev..

[44] Frewen P (2020). Neuroimaging the consciousness of self: Review, and conceptual-methodological framework. Neurosci. Biobehav. Rev..

[45] Stefanova E (2020). Anticipatory feelings: Neural correlates and linguistic markers. Neurosci. Biobehav. Rev..

[46] Morris JC (1989). The Consortium to Establish a Registry for Alzheimer's Disease (CERAD). Part I. Clinical and neuropsychological assessment of Alzheimer's disease. Neurology.

[47] Kynast J (2020). Age- and sex-specific standard scores for the reading the mind in the eyes test. Front. Aging Neurosci..

[48] Kynast J (2020). Mindreading from the eyes declines with aging—evidence from 1,603 subjects. Front. Aging Neurosci..

[49] Williams BW, Mack W, Henderson VW (1989). Boston Naming Test in Alzheimer's disease. Neuropsychologia.

