# Supplementary material for: Heart failure decouples the precuneus in interaction with social cognition and executive functions
Source: Sci Rep. 2023 Jan 23;13:1236. doi: 10.1038/s41598-023-28338-0 (PMC9870947; doi:10.1038/s41598-023-28338-0)
Supplement: Supplementary file 1 — Supplementary Legends. [file 41598_2023_28338_MOESM1_ESM.docx]

**Supplementary Figure S1: Precuneus connectivity decrease in heart failure (HF).** (**A**) Connectivity is decreased in HF in the precuneus using global correlation as a centrality measure (red color). (**B**) Using the precuneus in a seed-based correlation analysis, decreased precuneus connectivity was found with lower cognitive performance in HF. In particular, an interaction between the factors HF and cognitive performance was obtained showing a significant group difference (HF vs. no-HF) with respect to decreased precuneus connectivity with lower cognitive performance in HF (red color). The Figure illustrates the results using the threshold-free cluster enhancement (TFCE) toolbox (rows 1 and 3) and the LISA approach (rows 2 and 4). Both procedures were performed with 10,000 permutations using an alpha level of 0.05 (TFCE with family-wise error correction; LISA with false discovery rate). x, y, z - coordinates of the Montreal Neurological Institute (MNI) stereotactic space. L - left, R - right.
