# Supplementary material for: Heart failure decouples the precuneus in interaction with social cognition and executive functions
Source: Sci Rep. 2023 Jan 23;13:1236. doi: 10.1038/s41598-023-28338-0 (PMC9870947; doi:10.1038/s41598-023-28338-0)
Supplement: Supplementary file 2 — Supplementary Figure 1. [file 41598_2023_28338_MOESM2_ESM.pdf]

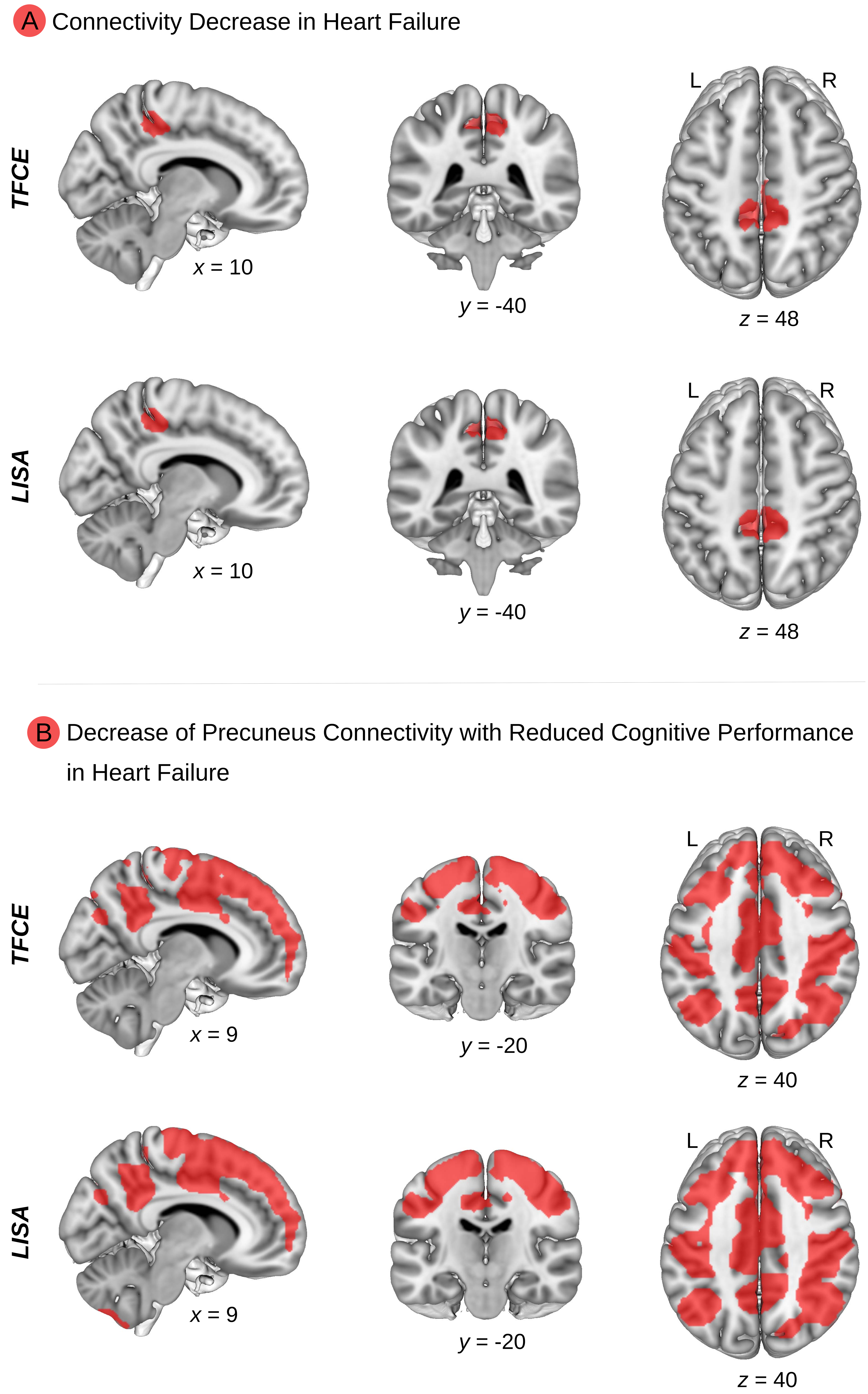
A Connectivity Decrease in Heart Failure
TFCE
LISA
x = 10
y = -40
z = 48
L
R
x = 10
y = -40
z = 48
L
R
B Decrease of Precuneus Connectivity with Reduced Cognitive Performance in Heart Failure
TFCE
LISA
x = 9
y = -20
z = 40
L
R
x = 9
y = -20
z = 40
L
R
